# Consistency Analysis of Redundant Probe Sets on Affymetrix Three-Prime Expression Arrays and Applications to Differential mRNA Processing

**DOI:** 10.1371/journal.pone.0004229

**Published:** 2009-01-23

**Authors:** Xiangqin Cui, Ann E. Loraine

**Affiliations:** 1 Section on Statistical Genetics, Department of Biostatistics, University of Alabama, Birmingham, Alabama, United States of America; 2 Department of Bioinformatics and Genomics, North Carolina Research Campus, University of North Carolina at Charlotte, Charlotte, North Carolina, United States of America; University of Pennsylvania School of Medicine, United States of America

## Abstract

Affymetrix three-prime expression microarrays contain thousands of redundant probe sets that interrogate different regions of the same gene. Differential expression analysis methods rarely consider probe redundancy, which can lead to inaccurate inference about overall gene expression or cause investigators to overlook potentially valuable information about differential regulation of variant mRNA products. We investigated the behaviour and consistency of redundant probe sets in a publicly-available data set containing samples from mouse brain amygdala and hippocampus and asked how applying filtering methods to the data affected consistency of results obtained from redundant probe sets. A genome-based filter that screens and groups probe sets according to their overlapping genomic alignments significantly improved redundant probe set consistency. Screening based on qualitative Present-Absent calls from MAS5 also improved consistency. However, even after applying these filters, many redundant probe sets showed significant fold-change differences relative to each other, suggesting differential regulation of alternative transcript production. Visual inspection of these loci using an interactive genome visualization tool (igb.bioviz.org) exposed thirty putative examples of differential regulation of alternative splicing or polyadenylation across brain regions in mouse. This work demonstrates how P/A-call and genome-based filtering can improve consistency among redundant probe sets while at the same time exposing possible differential regulation of RNA processing pathways across sample types.

## Introduction

Expression arrays from Affymetrix contain hundreds of thousands of oligonucleotide probes grouped into functional units called probe sets, where each probe set is designed to measure the expression of a known or computationally-predicted mRNA target molecule. Affymetrix sells two commonly-used types of expression arrays: three-prime arrays in which probe sets are designed against the three-prime region of a single mRNA target, called a consensus sequence in Affymetrix parlance, and exon-focused arrays in which probe sets are designed to interrogate individual exons. Currently, the majority of mammalian expression data in the Gene Expression Omnibus (GEO) are from three-prime arrays, especially the mouse 430 and human u133 series, and these arrays continue to be used in individual labs as well as larger-scale projects, such as the Cancer Genome Atlas [1]. Exon arrays were developed more recently and have been marketed as being able to quantify changes in alternative splicing. Many groups have reported success using exon arrays in this way, while others have explored alternative designs in which probes span exon-exon junctions and interrogate individual splicing events. In this paper, we argue that the three-prime arrays, although they were not designed specifically for this purpose, may have some value in the study of alternative transcripts, thanks to the large number of redundant probe sets present on many of these arrays.

Redundant probe sets are probe sets that measure different regions of the same target gene. As described in their product literature, the Affymetrix probe set design procedures for the three-prime arrays attempt to generate probe sets for all known or inferred expressed sequences. As a result, many of the three-prime arrays contain a large proportion of redundant probe sets that interrogate potential alternative forms of transcripts arising from the same gene. In most cases, redundant probe sets reflect differential three-prime end processing, since probes have typically been selected from regions near the three-prime ends of the target transcripts. For example, D'mello et al. [2] compared human, mouse, and rat Affymetrix GeneChips probes and probe sets to polyadenylation sites predicted from ESTs. They concluded that alternative polyadenylation may affect expression analysis of a large number of target genes (13%–35%) on each array.

Probe set redundancy can cause problems for microarray data analysis when different probe sets addressing the same gene produce inconsistent results. For example, if only one of a set of redundant probe sets appears differentially-expressed, should this lessen confidence that the gene itself is changed in response to the treatment being investigated in an experiment? How should an investigator interpret a scenario where multiple probe sets appear differentially-expressed, but in opposite directions? Such questions have largely been overlooked in most real-world analyses, mainly because commonly-used analysis protocols operate at the level of the probe set and incorporate probe set-to-gene annotations late in the process. It is not clear how how an investigator should interpret redundant probe sets that generate discordant results, which is one of the problems we address in this paper.

The most biologically-interesting and straightforward explanation for inconsistency among redundant probe sets is that the experimental condition under investigation has induced a change in the relative concentration of mRNA variants detected by the discordant probe sets. However, this interpretation is clouded by the fact that some redundant probe sets are not intended to measure variants of the same gene. For example, some redundant probe sets are merely designed to measure opposite strands of the same gene when the array design process was unable to determine the genomic strand of a transcribed sequence. In other cases, redundant probe sets map to the same strand of the same gene region, but their target sequences do not overlap and therefore are unlikely to be variants of the same transcriptional unit. For example, Stalteri and Harrison [3] described in detail the mouse *Surf4* gene that was associated with eight probe sets on the MOE430A chip. As described in their article, two out of the eight probe sets actually hybridize to transcripts arising from a different but related gene (*Surf2*) transcript. Our analysis of the redundant probe sets on the Arabidopsis ATH1 chip using a large collection of microarray data showed that some of the lack of concordance between the profiles from the redundant probe sets is likely associated with incorrect gene models and annotation problems [4]. Thus, the potential ability of redundant probe sets to shed light on regulation of mRNA variants is somewhat clouded by ambiguities in annotation, i.e., mapping probe sets onto their putative target genes.

Previously Affymetrix reported probe set-to-target gene mappings primarily as mappings between Unigene identifiers (ids) and probe set ids, reflecting the transcriptome-centric nature of the Affymetrix probe set design pipeline [5]. However, as genomic sequence has become available, Affymetrix has shifted toward a more gene-centric approach, reporting mappings between Entrez Gene ids and probe sets in addition to mappings between probe set ids and putative target transcripts. Because new sequences are constantly added to the public databases, the probe set-to-target mappings require constant updating to reflect the new data, and Affymetrix obliges this requirement via periodic new releases of probe set annotations. One group tracking these new releases showed that 5% of the Affymetrix probe set-to-gene annotation in Affymetrix' NetAffx database had changed over a two-year span [6]. Other groups besides Affymetrix have also tackled the problem of probe set annotation and target gene identification. Some of these efforts have helped to expose problematic or potentially faulty probes, such as probes that map to multiple locations in the genome or, conversely, probes that do not appear to map to any location within the designated target locus. Studies that have investigated problematic probes have demonstrated that removing them from estimates of target mRNA abundance profoundly affects analysis results [3], [7], [8].

In this paper, we investigate redundant probe set consistency in a data set harvested from the Gene Expression Omnibus [9]. We investigate the degree to which redundant probe sets, determined using default probe set annotations provided by Affymetrix, exhibit discordant results. We assess how genome-based and qualitative present/absent (P/A) screening methods affect probe set consistency, using different measures of differential expression. We use an ANOVA-based method to detect target genes whose redundant probe sets show significantly different fold-changes across experimental conditions. We then visually-inspect these target genes using an interactive genome display tool and determine if independent evidence for alternative splicing or polyadenylation is available. In general, we find that eliminating problematic probe sets through genome-based screening, followed by application of present/absent call filtering, results in an overall increase of consistency among redundant probe sets, leaving only the most interesting cases for further analysis and experimental verification.

## Methods

### Affymetrix-provided redundant probe set groupings

A file containing annotations for the Mouse 430_2 array was downloaded from Affymetrix. The file (Mouse430_2.na22.annot.csv) reports zero or more Entrez Gene ids for each probeset. Thus, probe sets mapping to the same gene id represent redundant probe sets according to Affymetrix' in-house annotation pipeline. Out of 45,101 probe sets listed in the file, 36,431 were listed as having one or more target genes. A group of probe sets that match the same target gene are designated redundant probe sets. (Note that MOE430 and 430_2 are alternative designations for the same array design.)

### Genome-based grouping and screening procedures

We obtained mouse genome (mm8) alignments for Affymetrix MOE430 probe set consensus sequences from the UCSC Genome Bioinformatics Table Browser in “bed” (browser extensible format) and fasta formats. The “bed” file describes the pattern of aligned blocks between matching segments of consensus and genomic sequence, and the fasta file contains concatenated segments of genomic sequence as defined by these alignment blocks. Probe sets with consensus alignments that mapped to a single genomic location within the assembled chromosomes were retained. We obtained probe sequences for the Mouse 430_2 array from Affymetrix and then computed the coordinates of each probe's position within the corresponding genome-based fasta sequences. Consensus alignments that contained all eleven probes were noted and carried forward for subsequent statistical analysis. Finally, we trimmed the alignments such that they included only the regions bounded by the five- and three-prime most probe positions. For visualization of the trimmed target regions and probes, we converted the trimmed alignments into Affymetrix' “link.psl” format and viewed them in the Integrated Genome Browser, an open source, freely-available interactive desktop genome browser tool (http://igb.bioviz.org). The “link.psl” file is available from http://www.transvar.org/results/reanal/MOE430.trimmed.link.psl.

In parallel, we obtained mRNA-to-genome alignments from the UCSC Genome Bioinformatics Web site using the Table Browser tool. mRNAs that mapped to a single genomic location were included in subsequent steps. The mRNA-to-genome and trimmed probe set consensus sequence alignments were then sorted into groups such that at least one alignment block from each group member overlapped with one or more blocks from at least one other alignment in the same group. The biological rationale for this is that alignment blocks in mRNA alignments represent exons, and when these exons overlap, this is reasonably good evidence that they originate from the same gene region or transcriptional unit. When a trimmed, probe set consensus sequence alignment overlaps with one or more mRNAs in a group, the probe set is then considered to interrogate the same gene region or transcriptional unit as defined by the mRNAs' pattern of alignment. When two or more trimmed, probe set consensus sequences belong to the same group, these probe sets are considered redundant because they measure the same gene region or transcriptional unit. To facilitate downstream analysis, Entrez Gene id-to-mRNA accession mappings were obtained from NCBI (the gene2accession file from the Entrez Gene ftp site) and then added to each gene grouping.

Data S1 lists groupings for screened probe sets, and Data S2 summarizes results from the screening steps. In the latter file, probe sets whose consensus sequences mapped to one or no genomic positions receive mapping codes “SM” or “NM” respectively, and probe sets mapping to multiple positions are annotated with code “MM.” For all “SM” probe sets, the number of probes perfectly matching the genomic sequence within the region defined by the probe set consensus alignment is reported.

### Microarray data set

Array data files (.CEL files) for expression microarray data set GSE4035 were obtained from the Gene Expression Omnibus. This experiment profiled the gene expression in the amygdala and hippocampus dissected from mice from two different strains that exhibit distinct responses to fear conditioning (high and low tolerance). Each brain region for each reaction level was represented by six biological replicates. For the redundant probe sets and differential expression analysis, brain regions were compared only between mice from the same strain [10]. We selected this data set in because of its relatively high level of replication (six arrays per group) and observations that alternative mRNA processing is unusually prevalent in neural tissue.

### Array pre-processing

Probe intensity data from each data set were imported into the R environment (http://www.R-project.org) directly from .CEL files using the *affy* package [11] in Bioconductor (http://www.bioconductor.org/). The *affy* package was also used to create expression summary measures. Briefly, we adjusted the background of perfect match (PM) probes, applied a quantile normalization of the corrected PM values, and calculated final expression measures using the robust multi-array average (RMA) method [12]. Pre-processing was performed on all chips in the data set together.

### Present/absent call filtering

In cases where Present/Absent filtering was conducted, P/A call for each probe set on each chip was obtained using the MAS 5.0 method implemented in the *affy* package with default settings unless specified. To be designated “present” in differential expression analysis, a probe set needed to have at least 80% of replicate samples called as present in at least one sample type.

### Probe set-level differential expression analysis

To estimate effects of each brain region and test for the region effect, we split the data according to fear reaction levels and then fit a model to the data from each probe set. For each subset of data we fit a fixed effect one-way ANOVA model, 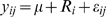, to the pre-processed expression level of each probe set. Here *μ* is the overall mean; *R_i_* is the deviation of the *i* ^th^ (i = 1,2) brain region from the overall mean; and *ε_ij_* is the residual. We used a shrinkage-based t test [13] to test the brain region effect (*R_i_*). The empirical distribution of the shrinkage-based t statistic was established through permutation analysis, where the rows of the design matrix corresponding to the tested term (*R_i_*) were shuffled 1000 times randomly while the data were kept unchanged [13], [14]. The shrinkage t statistics calculated from the permutations were pooled across genes that are not significant [15] at nominal 0.1 level according to a conservative gene specific t test to form one overall empirical distribution. The percentile of the shrinkage t from observed data in the empirical distribution provides an estimate of the p-value for each gene. Gene lists were generated using a false discovery rate (FDR) of 0.005 [16] unless otherwise specified.

### Comparing redundant probe sets

To determine when two redundant probe sets measuring the same gene generate discordant results, we formally tested whether the redundant probe sets produce different fold changes. One way to achieve this is to test whether the signal differences between redundant probe sets have a significant brain region effect. In other words, we compare the fold-change between tested groups (brain region, in this case) exhibited by two redundant probe sets and ask if they are significantly different. If two probe sets yield similar fold-changes, the differences will not show a significant group effect. If two probe sets generate different fold-changes, this indicates possible differential regulation of probe set targets. To test for different fold-changes, we first take the difference between the probe sets on the same chip after data pre-processing and then fit the ANOVA model used above for differential expression to the differences. The test of significance for the term of interest (*R_i_*) provides information on whether the targets of the two redundant probe sets are affected by this term differently. This method is a modified version of the ANOSVA methods by Cline *et al.* [17], adapted for pairwise probe set comparisons. The modification allows identification of specific pairs of disagreeing probe sets, a necessary first step toward identification of differentially-processed targets.

### Significance consistency index

We developed a significance consistency index of the redundant probe sets to summarize the overall degree to which redundant probe sets generate consistent results in a test for differential expression. This allows one to assess probe set-to-gene target annotations across the full set of probe sets in a given array. For a single group of redundant probe sets targeting *i^th^* gene *g_i_*, the consistency index was calculated as the proportion 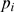 of redundant probe sets found to be significant with respect to differential expression. Only probe set groupings where there was at least one significant probe set were included in consistency index calculations. Averaging individual redundancy probe set group consistencies yields an overall consistency index 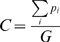, where 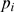 is the proportion of probe sets that are significant for gene *g_i_* (i = 1,…,G) and G is the total number of associated multi-probe set target genes being tested on an array in which at least one probe set was found to be significantly changed. Thus, values closer to one indicate greater overall agreement among redundant probe sets across all genes G represented on an array.

### Re-sample procedure assessing effects of sampling variation on probe set pair differences

We used a re-sampling approach to assess the effects of sampling variation on fold-change and P/A call consistency between redundant probe sets. To simulate variation arising from random sampling, we created sub-data sets from arrays in two different experiment groups: amygdala and hippocampus samples harvested from the low fear strain, where each group contained six replicate arrays. For this, we randomly-selected three arrays from each group, forming a single sub-data set of six arrays, including a three arrays from group one (S_1a_ - low fear amygdala) and three arrays from group two (S_1h_ - low fear hippocampus). The remaining six arrays formed a second sub-data set (S_2a_ and S_2h_). For each round of sampling, we assessed consistency using summary statistics comparing sub-data sets and redundant probe sets. We repeated the sub-setting procedure twenty times and calculated the average and variance for each measure. For simplicity, we considered probe sets with only one redundant partner probe set.

The summary statistics included: (1) PA-consistency for redundant probe sets, calculated as the percentage of redundant probe set pairs where each probe set in the pair had the same PA call in the two sub-data sets; (2) PA-consistency for single probe sets, calculated as the percentage of probe sets that had the same PA-call in the two sub-data sets; (3) Fold-change correlation for redundant probe sets, computed as the correlation of fold-changes pairs corresponding two redundant probe sets harvested from S_1_ and S_2_ array triplets, where fold-change (amygdala versus hippocampus) was calculated using array triplets from each sub-data set separately; (4) Fold-change correlation for individual probe sets, calculated as the correlation between fold-changes obtained from S_1_ versus S_2_; (5) No. of redundant probe sets pairs where both probe sets in the pair were significantly changed across brain regions (FDR 0.05), within the same sub-data set S_1_ or S_2_; (6) Number of individual probe sets that were significantly changed (FDR 0.05) across brain regions in both sub-data sets S_1_ and S_2_.

Note that one caveat to this random sampling approach is that the power to detect differential expression across brain regains test using the sub-data sets decreases is reduced in the sub-data sets because of the smaller number of replicates, e.g., reduced from six to three. Therefore, for this analysis, we adjusted the stringency for identifying differential probe sets to FDR 0.05.

## Results

Affymetrix provides annotation files mapping individual probe set onto Entrez Gene ids; these annotations are widely used in publicly-available databases (such as the Gene Expression Omnibus) and in microarray analysis software packages such as those in Bioconductor. These annotation files report Entrez Gene ids for many probe sets, and probe sets that are annotated with the same Entrez gene id represent redundant probe sets as determined by Affymetrix. Using matching of Entrez gene ids, we obtained a list of Affymetrix-designated redundant probe set groupings where all the probe sets in the same group match the same gene. These redundant probe set listings thus provide a baseline against which to evaluate improvements in probe set-to-target gene annotations. As shown in Table 1, the general distribution of the number of probe sets per gene is highly skewed. For example, groups with n probe sets are more than twice as common as groups with n+1 probe sets.

**Table 1:** Number of genes with redundant probe sets before and after filtering out the absent probe sets.

# of prs/gene	AG	AGP	GG	GGP
2	5,133	2864	3767	2337
3	2,427	1228	1173	677
4	1,181	496	353	205
5	556	256	105	55
6	305	99	25	10
7	144	35	7	3
8	69	18	1	0
9	30	4	0	0
10	15	0	0	0
11	7	0	0	0
12	4	0	0	0
13	3	0	0	0
15	1	0	0	0
Total # genes	9875	5000	5431	3287

AG and GG indicate the original Affymetrix grouping and genome-based groupings, respectively. AGP and GGP represent the AG and GG groupings in which only probe sets called as “Present” were included in the final groupings. The abbreviation “prs” means: “probe sets.”

**Figure 1:**
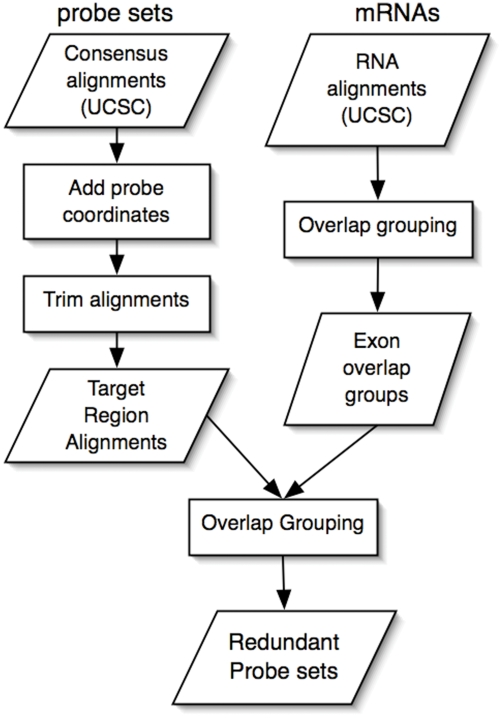
Scheme for genome-based redundant probe sets grouping. Genomic alignments for probe set consensus sequences are tested for instances of probe sequences within the genomic portion of the alignment. For all probe sets whose consensus sequence alignments contain all eleven probes, a new trimmed target region genomic alignment is generated bounded by the five and three-prime-most probe sequences. In parallel, genomic alignments for mRNA are combined into groups based on genomic overlap patterns. The trimmed target region alignments are then compared with the exon overlap groups from the mRNA branch of the pipeline. All probe set target sequence alignments that overlap with at least one member of the mRNA exon overlap groups are then annotated as targeting the given group. Probe sets interrogating members of the same exon overlap group are designated redundant probe sets.

We developed a genome-based computational pipeline that uses genomic alignments of mRNAs and probe set consensus sequences to identify high-quality probe sets and assign these to groups based on their patterns of genomic overlap. The goal of the pipeline is to generate a highly stringent set of annotations and make downstream, gene-by-gene analysis steps less perilous (Figure 1). Briefly, the method uses publicly-available genomic alignments to define probe set interrogation regions within the genomic sequence. Next, it searches for probe locations within the fasta sequence from concatenated genomic sequence defined by alignment blocks. Probe sets whose probes are omitted from the fasta sequence are flagged as questionable and omitted from the final list of “cleaned” probe sets. Using this procedure, we found that 94% of probe set consensus sequences on the Mouse 430_2 array mapped to a single genomic location. Of these, 86% contained all eleven probes in the genomic sequence.

We then used consensus and mRNA genomic alignments to sort the screened probe sets into redundant probe set groupings such that members of each group measure the same gene. Using this procedure, we identified around 5,000 genes or gene regions that were interrogated by two or more cleaned redundant probe sets for the MOE430 array. Compared with the groupings based on Affymetrix annotation, the groupings generated this way have many fewer genes with redundant probe sets (Table 1). Also, the group sizes tend to be smaller. The largest group contains 8 probe sets. In contrast, a substantial number of groups from Affymetrix' annotation file have more than 8 redundant probe sets, while others have as many as 15 probe sets.

### Redundant probe set consistency

Affymetrix microarray expression data are commonly analyzed probe set by probe set. If the redundant probe sets for a given gene indeed measure the same target transcripts, then they should yield consistent results in the same experiment with the allowance of some variation. We tested this expectation by examining present-absent call consistency, significance consistency, and fold change consistency across redundant probe sets in a relatively well-replicated, real-life data set (GSE4035) harvested from the Gene Expression Omnibus and generated using the mouse MOE430 array from Affymetrix. The data set consists of six samples per experimental grouping, where the groups consist of samples from different brain regions (amygdala and hippocampus) from two different strains of mice that exhibit differential fear responses. For differential expression analysis across sample types, we compared different brain regions dissected from the same mouse strain, thus avoiding complications due to genetic differences between strains.

### Consistency of present and absent calls

Affymetrix probe sets include perfect match (PM) probes that are identical to the probe set's intended target, as well as corresponding mis-match probes (MM) that contain a single base pair difference. The MM probes are believed to provide an estimate of non-specific hybridization that presumably affects the PM and MM probes equally. Comparing hybridization intensity of PM and MM probes allows assessment of the overall signal strength of a probe set. The low PM signals relative to MM probes can be identified using the qualitative, present/absent (P/A) call algorithms in the MAS5.0 software from Affymetrix [18]. The consistency of the P/A calls between probe sets interrogating the same gene provides an indicator of how differently the redundant probe sets from the same gene behave at this level. We ran the P/A call algorithm on all arrays and examined the consistency of P/A calls between redundant probe sets on the same array. We found that, on average, only about 50%–60% of probe sets are called as present, which is similar to the overall proportion of expressed genes in most tissues [19]. We found that redundant probe sets measuring the same gene (as designated by Affymetrix) frequently received different P/A calls. For example, genes with two redundant probe sets assigned by Affymetrix grouping showed inconsistent P/A calls in 31% of the genes. However, the redundant probe sets based on the genome-based groupings showed lower (only 26%) P/A call inconsistency. Overall, we found that P/A calls for the genome-based groupings were generally more consistent than for the Affymetrix groupings.

### Significance Consistency of redundant probe sets

Expression microarray data analyses from Affymetrix arrays generate lists of significantly-changed probe sets, and when one or more of a gene's matching probe sets appear in the list, then the target gene is typically considered to be differentially expressed. Because redundant probe sets may interrogate different transcripts arising from the same gene, examining the consistency of redundant probe sets that are included or excluded from the significant probe set list may expose biologically-interesting features of the data, such as evidence of differential mRNA processing. Using a relatively stringent significance level (FDR = 0.005) for selecting significant probe sets, we identified 4,982 and 8,952 significant probe sets that were differentially expressed between the amygdala and hippocampus samples in the low and high reaction levels in the GSE4035 data set. The overlap among these two lists is very high, with 4,041 probe sets in common. The fold changes obtained from these two reaction levels are also very similar (Data S3). For simplicity, we focused on the low reaction level alone for the significance consistency analysis. Among the 4,982 significant probe sets, we found that 3,193 probe sets were from genes that have two or more probe sets, as judged by our genome-based grouping and screening procedure. To determine the consistency of these redundant probe sets in terms of differential expression, we examined the presence or absence of all redundant probe sets in the significant list. We found that only a small proportion of the genes with at least one significant probe set have all probe sets significant, while the majority of them show inconsistent results from the redundant probe sets (Data S4). The results obtained from the high reaction level are similar to that of the low reaction level presented here.

**Figure 2:**
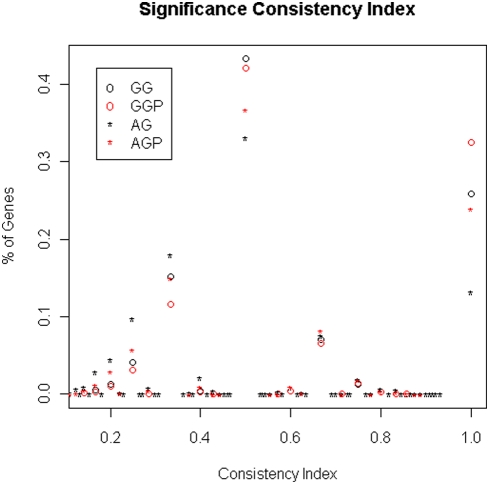
The distribution of genes with various significance consistency index values. A consistency index was calculated for each gene with multiple probe sets and at least one probe set significantly different across compared groups. The y-axis indicates the proportion of genes with the consistency index indicated on the x-axis. AG and GG refer to Affymetrix groupings and genome-based groupings, respectively. AGP and GGP refer to AG and GG groupings in which only probe sets called as “present” were included in the calculation. Significance level is FDR 0.005.

**Figure 3:**
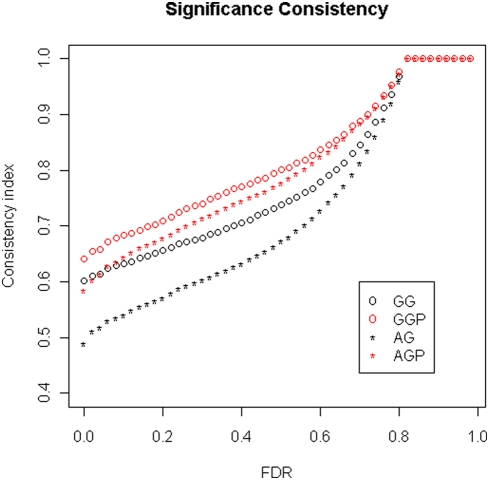
Significance consistency among the redundant probe sets changes depends on FDR threshold. A consistency index was computed for each gene with redundant probe sets at different FDR level. AG and GG indicate the original Affymetrix grouping and proposed genome-based groupings, respectively. AGP and GGP represent the AG and GG groupings. AGP and GGP refer to AG and GG groupings in which only probe sets called as “present” were included in the calculation.

To summarize the overall consistency of the redundant probe sets in terms of whether they are significant, we calculated the consistency index using genes with redundant probe sets where at least one probe set was identified as significantly-changed between compared groups. Figure 2 shows that the majority of the redundant probe set groupings have consistency index less than one. The large proportion of genes with consistency index of 0.5 or 1 is due to the fact that most genes in our groupings have only two probe sets and at least one of them is significant for this calculation (Table 1). Compared with the Affymetrix grouping scheme, the genome-based grouping has a larger proportion of genes showing higher consistency and smaller proportion of genes showing lower consistency. We also computed the average consistency score across all genes. It is 0.60 when genome-based grouping was used. However, it is 0.50 when the Affymetrix grouping was used. The larger consistency index obtained for the genome-based grouping indicates that this probe set screening procedure increases the consistency across redundant probe sets. Considering that the consistency index may change when different FDR thresholds are used for identifying differentially expressed probe sets, we also examined the overall consistency index at various FDR thresholds (Figure 3). We found that the consistency index increases as the FDR threshold decreases. However, regardless of the FDR threshold, the consistency index of the genome-based grouping exceeds that of the Affymetrix grouping, although the difference decreases with increasing FDR.

### Removing absent probe sets improves redundant probe set consistency

It was demonstrated previously that filtering out probe sets that are deemed as absent (P/A filtering) before conducting further statistical inference benefits identification of differentially expressed genes [20]. To test the effects of P/A filtering on redundant probe set consistency, we removed probe sets that were deemed as absent by MAS5.0 present/absent call [21] and only analyzed the redundant probe sets that were deemed present. When we recomputed the significance consistency index for the present redundant probe sets and excluded absent probe sets from the calculations, the consistency index increased dramatically. Figure 2 shows that removing the absent probe sets using P/A filtering increased the proportion of genes with higher consistency and decreased the proportions of genes with lower consistency. The significance consistency index for the genome-based grouping increased from 0.60 to 0.65 with P/A filtering. For the Affymetrix grouping, P/A filtering increased the consistency index from 0.50 to 0.59.

To further examine the relationship among P/A calls, significant probe sets, and consistency of the redundant probe sets, we examined in detail the genes with two probe sets. In general, probe sets that indicate differential expression are also called as Present by MAS5.0 (Table 2). For the 2,002 genes with both probe sets called as present (P/P category in Table 2), about 22% (884) of the probe sets were significant and more than 57% of the 884 significant probe sets were from the same genes. In contrast, for genes with both probe sets absent (A/A category), only three probe sets from the 834 genes were significant and each of these three significant probe sets were from different genes. For genes with one present probe set and one absent probe set (P/A category), 9.6% (178) of the probe sets from the 931 genes were significant and 13% of the significant probe sets were from the same gene. In addition, the significant probe set is the present probe set in most cases (147 out 154). These results indicate that the significant probe sets and the significance consistency among redundant probe sets come mainly from the present probe sets, which explains why removing absent probe sets improves the overall consistency of redundant probe sets. We also formally tested whether the proportions of significant probe sets in each of the three categories in Table 3 (P/P, P/A, A/A) are different using a McNemar test [22] and obtained extremely high significance (with p values less than 2.2×10^−16^). The same test was also conducted to test the proportions of significant probe sets that are from the same gene across the three categories (P/P, P/A, A/A), and the result was also extremely highly significant (with p values less than 2.2×10^−16^). These tests confirm that absent probe sets indicate differential expression to a lesser degree than do present probe sets and that removing absent probe sets increases redundant probe set agreement.

**Table 2:** Relationship between P/A calls and significance for differential expression for genes with two probe sets.

	P/P	A/A	A/P
Total # genes	2002	834	931
Sig probe sets	884	3	178
2 (1) sig prs/gene	253 (378)	0 (3)	12 (154)

P/P, both probe sets are present. A/A, both probe sets are absent. A/P, one probe set is absent and the other one is present. The abbreviations “sig” and “prs” mean “significant” and “probe sets,” respectively.

**Table 3:** Consistency of fold changes between redundant probe set pairs.

Statistics	AG	AGP	GG	GGP
Corr. Coef. of FC	0.60	0.74	0.72	0.79
% genes w/ opposite FC direction	0.38	0.28	0.33	0.27

AG and GG indicate the Affymetrix grouping and genome-based groupings, respectively. AGP and GGP represent the present the AG and GG groupings and including only present probe sets. The abbreviation “prs” means probe sets. FC is fold change. Corr. Coef is Pearson correlation coefficient.

The default stringency setting of the P/A call criteria used above was meant to control the false present calls rate at 0.05, i.e., an expected false present call rate of 5%. At this rate, the false absent call could be high. We increased the false present call rate to 0.1, 0.3, and 0.5 and examined how these different rates affected consistency of the redundant probe sets called as present. The overall proportion of present probe sets increased dramatically from 54% to 82% as the false present call rate increased. The P/A call consistency between redundant probe sets increased from 75% to 85% for genes with two probe sets. However, the overall significance consistency index for the present redundant probe sets reduced slightly from 0.65 to 0.61 (Data S5). This trend is observed for all the FDR settings in differential expression analysis (Data S6). These results showed that increasing the false present call rate resulted in large increase of the number of present probe sets and large increase of P/A call consistency with a small decrease of the significance consistency. It suggests that a higher P/A call threshold might be more appropriate in routine Affymetrix expression array data analyses.

### Fold change consistency among redundant probe sets

Fold change estimation is an important aspect of microarray analysis. It has been suggested that fold change is more replicable than p-value based inferences and often has greater biological meaning [19]. Therefore, we evaluated correlation between the fold changes obtained from redundant probe sets, focusing as before on genes with two redundant probe sets (Table 3). We found that the Pearson correlation coefficients were higher from the genome-based redundant probe sets (0.72) than from the Affymetrix-defined redundant probe sets (0.60). In addition, P/A filtering increased fold-change correlations. We also examined the proportion of genes in which the redundant probe sets exhibited fold-changes in the opposite direction and found that the Affymetrix-defined redundant probe sets had a higher percentage of opposite-directed fold-changes (38%) than the genome-based redundant probe sets (33%), and this difference was reduced by filtering out the absent probe sets. Thus, we conclude that the genome-based grouping in combination with P/A filtering improved fold-change correlation among redundant probe sets and also reduced the proportion of genes whose redundant probe sets indicated opposite fold change directions.

**Figure 4:**
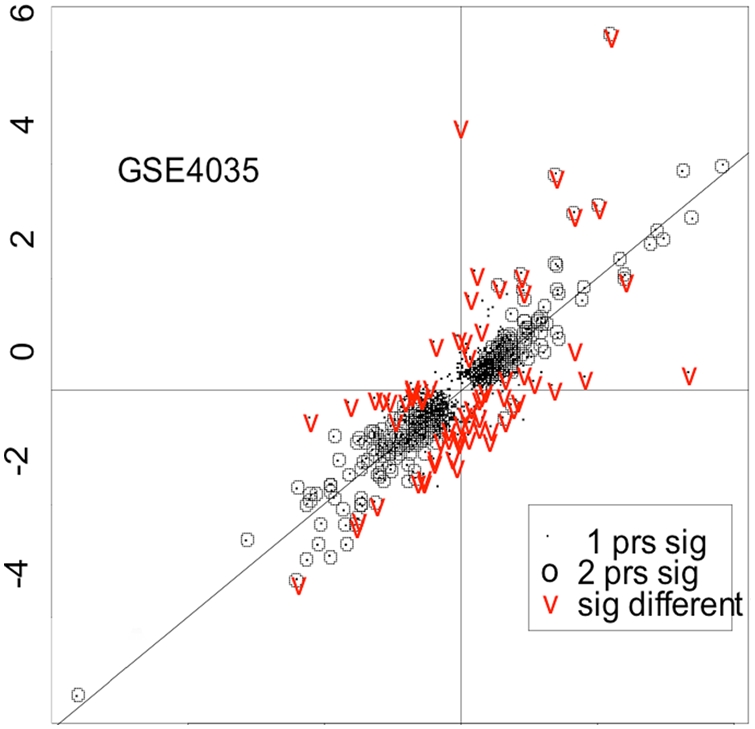
Fold change comparison across redundant probe sets. We first tested each probe set on the array for the brain region effect and identified a list of significant probe sets using FDR 0.005 significance level. We then compared this significant probe set list with the list of genes with two present redundant probe sets according to genome based grouping. For genes with at least one significant probe set, we plotted the two fold changes (on log scale) against each other with random naming of probe set1 (prs1) and probe set2 (prs2) in a gene. Genes with one significant probe set are presented by points. Genes with two significant probe sets are represented by circles. We also tested the two fold changes obtained from each probe set for genes with two redundant probe sets using FDR 0.05 significance level as described in Methods. Genes that show significant difference in their fold changes are represented by red V.

The above fold-change consistency analysis does not consider the whether a gene is significantly changed in an experiment. However, investigators are typically more interested in genes that are significantly-changed as a result of an experimental condition, tissue source, or other factor. To evaluate the fold-change consistency for genes in the significant list, we computed the fold-change correlation coefficient for genes with two present redundant probe sets such that at least one of these was significantly different between the two brain regions. The resulting correlation coefficient of fold-changes for this subset of redundant probe set pairs is 0.84, which is substantially higher than the correlation obtained from all genes with two present redundant probe sets (0.79). Only a very small proportion of probe set pairs (0.09%) in this group showed opposite fold change directions. These results suggest that the redundant probe sets from highly significant genes are generally more consistent. Figure 4 summarizes these results in graphical form.

P/A filtering stringency also affected the correlation of fold changes from redundant probe sets. For the present redundant probe sets, the correlations between the fold changes showed reduction (0.79 to 0.74) as P/A call p value threshold increased from 0.05 to 0.5. The proportion of genes with opposite fold change directions also increased from 27% to 30% (Data S5). These results provide evidence that the decrease of P/A call stringency also reduces the fold change consistency among the redundant probe sets called as present.

### Comparing consistency between and within redundant probe sets

Although redundant probe sets are expected to be consistent if they are interrogating the same transcript in a data set, random sampling will always introduce some variation. Thus, lack of consistency between redundant probe sets could arise from variation due to sampling, rather than a change in the relative concentration of mRNA targets. To evaluate the plausibility of random variation due to sampling as a potential cause of the observed discordance between redundant probe sets, we performed a re-sampling procedure aimed at simulating variance due to sampling and then evaluated redundant probe set concordance across the samples. For this, we separated the six replicates in each brain region (amygdala and hippocampus) of the low tolerance group into two sub-data sets, containing three replicates each from the two brain regions. The consistency of the results obtained from the same probe set compared to itself across the sub-data sets thus provides a measure of the variability introduced by random sampling. Comparing this sampling-induced variability with the variability observed between redundant probe sets within the same sub-data set then allows an assessment of whether or not the observed differences between redundant probe sets are due to sampling variability alone. For example, if the underlying target molecule differences between two redundant probe sets do not contribute much to their observed inconsistency, then the relative consistency for a single probe set's behaviour across the two sub-data sets should be similar in magnitude to the consistency observed between two redundant probe sets. In other words, we are asking whether redundant probe sets behave as replicates of each other in the re-sampling scenario.

For each of twenty repeated re-samplings, we calculated consistency with respect to PA calls, fold-change correlation, and differential expression twenty times, as described in Methods. The average and standard deviation of the values obtained appear in Table 4. The results show that P/A calls for single probe sets are extremely consistent across sub-data sets; we found that, on average, around 95% of probe sets are called as either Present or Absent in subsets taken from the same brain region. In contrast, only 60% of redundant probe sets are both called as Present or Absent. We observe a similar pattern with respect to fold-change correlation. Average fold-change correlation for individual probe sets calculated across sub-data sets was 0.956, while average correlation for redundant probe sets was 0.860. We also examined the number of genes in which both redundant probe sets were significant in the same test versus the number of probe sets significant in both subset data tests. The average number of genes with same probe set significant in both tests is 380 while the number of genes with both redundant probe sets significant in both tests is 301. In every simulation, the former was larger than the latter. These results indicate that the consistency across tests of different sub data sets for a single probe set is much higher than that between redundant probe sets within a test. The inconsistency is probably largely due to the nature of the redundant probe sets and their targets rather than due to random experimental variation.

**Table 4:** Comparison of consistency across sub data sets and consistency across redundant probe sets based on re-sampling.

Calculation method (see Methods)	Summary statistic	Average	Std. Dev.
1	PA consistency – redundant probe sets	0.608	0.130
2	PA consistency – same probe set across sub-data sets	0.947	0.018
3	Fold-change correlation – redundant probe sets	0.874	0.009
4	Fold-change correlation – same probe set, across sub-data sets	0.956	0.0094
5	# of redundant probe sets, with both members of the pair significantly different within a sub-data set	301.1	61.4
6	# of probe sets, significantly different in both sub-data sets	380.1	76.4

The subset of data corresponding to low reaction level were divided into two data sets by randomly sampling 3 out of the 6 biological replicates from each brain region. Each probe set was individually and with its redundant probe set partner for differential expression, fold-change correlations across and within sub-data sets, as described in the Methods. The mean and standard deviation were calculated for each summary statistics across twenty re-samplings of the data.

### Redundant probe sets that give significantly different fold-changes

If redundant probe sets show very different response to the treatment or condition, interesting biological phenomena, such as splicing or polyadenylation variants that respond to treatment or condition differently, may be revealed. Therefore, it is interesting to identify those redundant probe sets for further exploration. To achieve this, we used an ANOVA model (described in Methods) to examine some redundant probe set pairs for which both probe sets in the pair received a MAS5 “Present” call. Using a shrinkage-based t test, we found that 70 probe set pairs that showed significantly-different fold-changes between the two brain regions relative to each other (Figure 4). Interestingly, many of these pairs included probe sets that were not found to be themselves differentially-expressed across brain regions. These results suggest that even if one or both probe sets in a redundant pair do not suggest differential expression in isolation, together they may indicate a condition-dependent change in the relative concentrations of individual probe set target transcripts.

We examined the 70 genes with significantly different fold changes between the two redundant probe sets using the Integrated Genome Browser (IGB), an interactive genome visualization tool that can show probes and probe sets together with genomic sequence and sequence annotations (http://igb.bioviz.org). Using the Browser, we examined the probe target regions and compared these to the knownGene annotations harvested from the UCSC Genome Informatics Table Browser. We found that 29 of the 70 probe set pairs appeared to distinguish between different transcript variants according to knownGene mRNA annotations. In these cases, the relative expression of alternative transcript forms could differ across brain regions. In 5 cases, probe sets did not appear to overlap with any known genes or were associated with a pair of mRNAs that overlapped only across their 3-prime and 5-prime regions and therefore unlikely to be transcribed from the same promoters. (Note that mRNAs with this configuration are typically associated with different Entrez Gene ids; our scheme flags such cases where redundant probe sets are grouped together with mRNAs associated with different Entrez Gene ids as “mixed overlap groups.” For a list of these, see Data S1.) In another 4 cases, the probe sets target very small genes whose known transcripts encompass one or no introns. The remaining 32 redundant probe set pairs were associated with only one knownGene mRNA; one possible explanation for this result is that there may be some additional variant forms that have not yet been discovered or not yet recorded in the knownGene collection (Data S7).

Redundant probe sets, by definition, interrogate different regions of the same gene. As a result, they hybridize to different locations along the same transcripts. Except in cases where probe sets overlap along the transcript, the difference in the fold changes revealed by different probe set could also be due to the different degrees of degradation at different regions of targeted transcripts. To test this possibility, we examined the RNA degradation controls on the Mouse430v2.0 chip (*Gapdh*, β-Actin, *TransRec*, and *PyruCarb*). Each of these control genes has 3 to 4 probe sets located at the 5′end, middle, or 3′ end of a single target transcript. We tested adjacent probe sets and the probe sets at the two ends for fold change difference across groups using the same procedures used above in both data sets. No comparison was significant at the nominal 0.05 level in either data set. This result indicates that RNA degradation is not likely a major explanation for the different fold changes across groups exhibited by redundant probe sets in some genes as shown in Figure 4.

## Discussion

Redundant probe sets can complicate analysis of gene expression, but they may also yield information about how the relative concentrations of individual transcript variants change in response to diverse conditions or treatments. However, taking advantage of redundant probe sets in this way first requires a careful examination of probe set quality and annotations. We used genomic alignments for the probe set consensus sequences and their pattern of overlap with reference mRNA-to-genome alignments provided by UCSC Genome Bioinformatics to screen and group redundant probe sets such that only probe sets with reliable genomic mappings remained. This process excluded potentially-flawed probe sets and, at the same time, grouped the remaining probe sets into clusters of redundant probe sets based on their mutual overlaps with mRNA-to-genome alignments harvested from the “knownGene” track of the UCSC Genome Browser.

We tested these genome-based groupings by comparing them to the Affymetrix-provided redundant probe set groupings in a differential expression analysis of the GSE4035 data set, which involved comparisons of gene expression between two different brain regions in the mouse. Table 5 provides a summary of several consistency metrics we evaluated. To summarize consistency among redundant probe sets measuring the same gene, we developed a simple metric, the consistency index, defined as the percentage of probe sets measuring a single gene that are called as differentially-expressed in an experiment, averaged over all genes with multiple probe sets. We found that the genome-based redundant probe set groupings had a higher overall consistency index when compared to the Affymetrix groupings. As a further measure of redundant probe set consistency, we computed Pearson's correlation coefficient between the fold-changes obtained for the two different probe sets belonging to each redundant probe set pair. As with the consistency index, fold-change correlation was higher for genome-based groupings. In all cases, fold-change correlation and consistency index improved when only probe sets called as “Present” in 80% or more samples in a group were considered.

**Table 5:** Summary of redundant probe sets consistency evaluation.

Evaluated Metric	Groups Compared	Statistics Used
P/A call	Redundant probe sets from genes with just two redundant probe sets	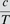 c, the number of genes with consistent P/A call; T, the total number of genes analyzed
	Random biological samples of the same probe set	
Significance for brain region effect	Redundant probe sets for all genes with redundant probe sets	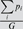 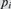 , the proportion of probe sets that are significant for gene i (i = 1,…,G); G, the total number of associated multi-probe set target genes being tested on the array.
	Present redundant probe sets for all genes with redundant probe sets	
	Random biological samples of the same probe set	
Fold change	Redundant probe sets from genes with two redundant probe sets	Pearson correlation coefficients and proportion of genes with opposite signed fold changes
	Present redundant probe sets from genes with two redundant probe sets	
	Present redundant probe sets from genes with two redundant probe sets and at least one probe set significant	
	Random biological samples of the same probe set	

We conclude based on these results that the genome-based grouping and screening method yields more consistent results for redundant probe sets. By grouping probe sets based on their genomic overlaps with known mRNAs, we obtained a more realistic picture of redundant probe set targets. And by only considering probe sets whose target regions map precisely to a single location in the genome which in turn contains perfect matches for all eleven probes, we help to rule out doubts that inconsistencies observed between redundant probe sets are due to cross-hybridization or probe set-design artefacts. Thus, the genome-based groupings have the potential to reveal biologically-interesting differential regulation of mRNA processing. To demonstrate this latter idea, we showed how a simple ANOVA-based method can detect significantly-different fold-changes for redundant probe sets that measure the same gene. Our application of the model identified redundant probe set pairs where the probe sets within a pair yielded significantly-different fold-changes. This method can be easily extended for genes with more than two redundant probe sets using a two-way ANOVA model as proposed by Li et. al., [23]. In the two-way ANOVA model, brain regions and probe sets are the two factors, and the interaction term of these two factors will capture the relative target level change associated with brain region. This two-way ANOVA model differs from the analysis presented here in that the response variable is the probe set intensity after normalization, while in the current analysis it is the difference between redundant probe sets, for genes with only two redundant probe sets.

Because we applied the test to redundant probe sets as defined by the genome-based screening and grouping method, we can be more confident that differences they detect are not mostly due to mis-annotations but instead may represent bona fide cases of condition-dependent differential mRNA processing. When we inspected the gene structures and probe set consensus sequence alignments using the Integrated Genome Browser, we found that about half of our findings have redundant probe sets interrogating different target mRNAs based on the knownGene transcript forms.

Alternative approaches to probe set redundancy have been developed. One seeks to avoid the complexity of the redundant probe sets by redefining Affymetrix probe sets in custom Chip Definition Files (CDFs), so that all probes from redundant probe sets are assembled into a single probe set [24]. In this case, the analysis is simplified to just analyzing one (potentially enormous) “mega probe set” per gene. It has been shown that using these custom CDFs can improve reproducibility of differential expression analysis between different laboratories [25]. However, consolidating the redundant probe sets eliminates the potential ability for individual probe sets to detect distinct transcripts. Redundant probe sets exist because the Affymetrix probe set design pipeline for three-prime arrays is capable of recognizing and handling transcript variants. The consolidation strategy perhaps allows for improvements in gene-level differential expression analysis, but it may eliminate any potential for detecting bona fide differential regulation of alternative transcripts, e.g., cases where the treatment under investigation changes the ratio of alternative forms expressed by the same transcriptional unit. Similarly, it is not clear if these “mega probe sets” have excluded probes from problematic probe sets, such as the probe sets interrogating incorrect strand as described for *Surf4* [3].

Another strategy is to sub-divide the probe sets into smaller groups of probes that that interrogate individual exons using prior knowledge of sequences present in transcript databases [26]. This strategy tends to generate a large number of probe sets containing fewer probes. Although this strategy has great appeal, simulation studies showed that results obtained from probe sets with fewer than four probes are not reliable, and therefore should be removed from an analysis. Any remaining probe sets are expected to detect different transcripts or clusters of transcripts and would therefore provide transcript-level differential expression measurements. However, because a large number of rare transcripts (in rare tissues and conditions) are considered in the same way as common transcripts, most of the probe sets may not detect particular transcripts in each experiment. The division of the probe sets may reduce the power for overall gene expression comparison.

Both the sub-division and consolidation strategies generate probe sets of unequal sizes, the influence of which on expression analysis is unknown. While both ideas have some appeal, we would argue that the probe sets, although some are flawed, capture real information about sequence variants that existed in the public databanks at the time the probe sets were designed. For the purposes of detecting condition-dependent differential mRNA processing of known variant forms (not detecting unknown variants), we recommend retaining the Affymetrix defined probe sets while at the same time depending on the high-quality ones for more detailed and careful analyses. Similarly, only probe sets that are truly redundant (e.g., hybridize with transcripts arising from the same gene or gene region) should be used to address alternative mRNA processing in Affymetrix 3-prime arrays.

Another recently-published approach involves treating redundant probe sets as replicate measurements of the same gene in an attempt to improve statistical power in differential expression inference [23]. This approach respects the original design of the array, and uses a statistical model to incorporate measurements from all redundant (termed sibling) probe sets, but only when the probe sets behave similarly in an experiment based on the insignificant result from testing the interaction term. In their article presenting the approach, Li and co-workers showed that their strategy has greater power to detect differentially expressed genes than individual probe set analysis or the consolidated “mega probe set” strategy. Their work differs from ours in that we focus on the differences between redundant probe sets, aiming to identify candidates for regulated alternative mRNA processing. Li et al focus on detecting differential expression of the entire target gene and use redundant probe sets only when they respond the same way to an experimental treatment. In general, most approaches that attempt to handle redundant probe sets are aimed either at using them to improve gene-level inference or detect novel splice variants. Our approach, by contrast, aims to detect candidates for differential regulation of mRNA processing, using what is already known about the genomic landscape and respecting the original Affymetrix design, which, although imperfect, reflects the known universe of mRNA transcripts.

In addition to probe set-to-target-gene mappings, Affymetrix assigns a grade to each probe set according to how well it matches a designated target transcript, independent from the Entrez Gene annotations. The probe set grades are based on sequence analysis comparing probes to their annotated targets. Although valuable, they do not specifically address the redundancy of probe sets but instead describe how well a probe set's constituent probes match a transcript (mRNA) record. For the purposes of redundant probe set assignments and examining alternative splicing/polyadenylation, it is better to examine the exon-intron structure of target mRNAs via their genomic alignments, since ultimately these are what biologists and data analysts will use to assess whether a given pair of probes sets interrogate an alternatively spliced or polyadenylated gene.

It is important to note that Affymetrix continues to develop new array designs, such as exon-focused arrays in which probes are selected from all known exons, and, more recently, reduced genome exon arrays which query all known exons but use fewer probes per target gene. However, the continuing popularity of the three-prime arrays and availability of large amounts of archived expression data in resources such as the Gene Expression Omnibus argue in favour of continued exploration of new analysis methods. The redundant probe sets on the three-prime arrays offer a means to measure differential three-prime end processing, e.g., alternative terminal exon choice and alternative polyadenylation. We expect that in the future, the exon arrays (or specialized splicing arrays) will be the tool of choice to measure differential mRNA splicing, while data from the three-prime arrays will get a second life as a rich source for data-mining alternative three-prime end processing mechanisms. Ultimately, the analysis procedures demonstrated here can be applied to multiple data sets to reveal large-scale patterns of alternative mRNA production and regulation.

## Supporting Information

Data S1 Genome-screened redundant probe sets. This file reports Entrez Gene targets (when available) and genome-screened redundant probe sets. Each row lists a set of redundant probe sets. Column 1 gives the Entrez Gene id; column 2 gives a GenBank accession for a mRNA overlapping the region; column 3 reports the group type; and column 4 contains a comma-separated list of redundant probe set ids. All probe set ids are from the mouse MOE430_2 array from Affymetrix. Group type (column 3) designation “overlap” indicates that the probe sets all match the same Entrez Gene. Group type “mixed_overlap” indicates that some probe sets within a group map onto more than one Entrez Gene id. This can occur when a first exon in one gene overlaps the last exon in another.
(0.28 MB TXT)

Data S2 Probe set screening results. This tab-delimited spreadsheet lists every probe set on the mouse 430_2 array from Affymetrix. Column 1 gives the name of the probe set; column 2 (labeled “map”) reports whether the probe set maps to one (SM), more than one (MM) or no (NM) locations in the mm8 mouse genome assembly; and the third column (labeled “probes”) reports the number of probes per probe set that map to the genome for all probe sets designated SM in column 2.
(0.78 MB TXT)

Data S3 Plots Showing Differentially Expressed Probe Sets. This file contains plots summarizing differential expression analysis results for the two subsets of data from GSE4035. Each fear condition level was treated as one data set after data pre-processing. The two brain regions were compared to identify differentially-expressed probe sets.
(0.06 MB DOC)

Data S4 Redundant Probe Set Consistency with Respect to Differential Expression. This file (divided into four sections) reports the number of significant probe sets for genes interrogated by redundant probe sets established using different grouping methods with or without removing the absent probe sets. Only genes with at least one differentially-expressed probe set are included.
(0.00 MB TXT)

Data S5 Effects of Varying Present/Absent Alpha Level. This file reports the effects of altering P/A call expected false positive alpha rate settings on redundant probe set consistency.
(0.01 MB XLS)

Data S6 Significance Consistency Results. Plots illustrating how significance consistency varies with respect to the false positive alpha rate setting for P/A calls.
(0.06 MB DOC)

Data S7 Gene Structure Inspection Results. Results from manual inspection of gene structures using the Integrated Genome Browser for the 70 genes with two present redundant probe sets that show significant different fold changes between two brain regions.
(0.03 MB XLS)
