# Supplementary material for: Consistency Analysis of Redundant Probe Sets on Affymetrix Three-Prime Expression Arrays and Applications to Differential mRNA Processing
Source: PLoS One. 2009 Jan 23;4(1):e4229. doi: 10.1371/journal.pone.0004229 (PMC2621337; doi:10.1371/journal.pone.0004229)
Supplement: Data S3 — Plots Showing Differentially Expressed Probe Sets. This file contains plots summarizing differential expression analysis results for the two subsets of data from GSE4035. Each fear condition level was treated as one data set after data pre-processing. The two brain regions were compared to identify differentially-expressed probe sets. (0.06 MB DOC) [file pone.0004229.s003.doc]

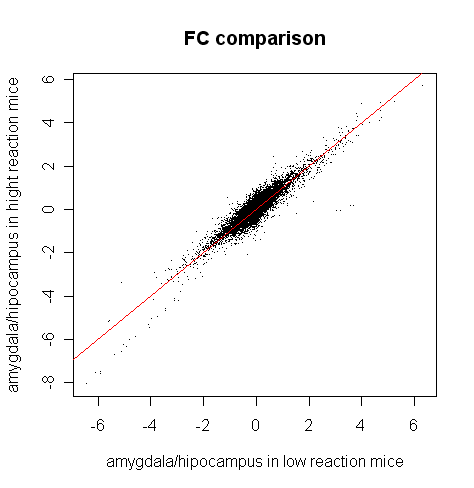


Fold change comparison between the two subsets of data corresponding to the two fear conditioning levels. The fold changes are on log2 scale. Red line is the identity line.


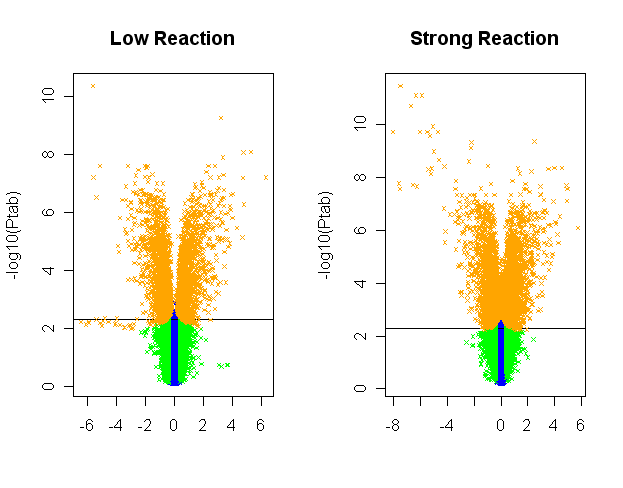


Volcano plots from testing between the two brain regions in each subset of data. The x axis is the fold change between amygdale and hippocampus on log2 scale. The left panel is from the low fear conditioning level and the right panel is from the high fear conditioning level. Probe sets above the horizontal line are significant based on conventional t testing. Orange points indicate significant genes based shrinkage-based t test. The green points are significant based t test with the assumption that the all probe sets have the same variance. Significance level is FDR 0.005.
