# Supplementary material for: Consistency Analysis of Redundant Probe Sets on Affymetrix Three-Prime Expression Arrays and Applications to Differential mRNA Processing
Source: PLoS One. 2009 Jan 23;4(1):e4229. doi: 10.1371/journal.pone.0004229 (PMC2621337; doi:10.1371/journal.pone.0004229)
Supplement: Data S6 — Significance Consistency Results. Plots illustrating how significance consistency varies with respect to the false positive alpha rate setting for P/A calls. (0.06 MB DOC) [file pone.0004229.s006.doc]

The significance consistency varies along the setting of the alpha of the P/A call function in the *affy* package. The alpha for the first to the forth picture is 0.05, 0.1, 0.3, 0.5 in each set. These settings are from very stringent to very liberal. Pictures 1 to 4 are the frequency counts for different consistency index. Pictures 5 to 8 are the overall significance consistency that varies along FDR settings.

Below is the consistency along FDR change with different P/A alpha level
